# JAK2^V617F^ myeloproliferative neoplasm eradication by a novel interferon/arsenic therapy involves PML

**DOI:** 10.1084/jem.20201268

**Published:** 2020-10-19

**Authors:** Tracy Dagher, Nabih Maslah, Valérie Edmond, Bruno Cassinat, William Vainchenker, Stéphane Giraudier, Florence Pasquier, Emmanuelle Verger, Michiko Niwa-Kawakita, Valérie Lallemand-Breitenbach, Isabelle Plo, Jean-Jacques Kiladjian, Jean-Luc Villeval, Hugues de Thé

**Affiliations:** 1 Institut National de la Santé et de la Recherche Médicale (INSERM) U1287, Gustave Roussy, Villejuif, France; 2 Université Paris-Saclay, Gustave Roussy, Villejuif, France; 3 Gustave Roussy, Villejuif, France; 4 Laboratoire d’Excellence GR-Ex, Paris, France; 5 Département d'Hématologie, Gustave Roussy, Villejuif, France; 6 Université de Paris, INSERM UMR-S1131, Institut de Recherche Saint-Louis (IRSL), Hôpital Saint-Louis, Paris, France; 7 Service de Biologie Cellulaire, Assistance Publique Hôpitaux de Paris (APHP), Hôpital Saint-Louis, Paris, France; 8 INSERM U944, Centre National de la Recherche Scientifique (CNRS) UMR7212, IRSL, Hôpital Saint-Louis, Paris, France; 9 Collège de France, Paris Sciences et Lettres Research University, INSERM U1050, CNRS UMR7241, Paris, France; 10 Centre d'Investigations Cliniques, APHP, Hôpital Saint-Louis, Paris, France; 11 Service de Biochimie, APHP, Hôpital Saint-Louis, Paris, France

## Abstract

Interferon α (IFNα) is used to treat JAK2^V617F^-driven myeloproliferative neoplasms (MPNs) but rarely clears the disease. We investigated the IFNα mechanism of action focusing on PML, an interferon target and key senescence gene whose targeting by arsenic trioxide (ATO) drives eradication of acute promyelocytic leukemia. ATO sharply potentiated IFNα-induced growth suppression of JAK2^V617F^ patient or mouse hematopoietic progenitors, which required PML and was associated with features of senescence. In a mouse MPN model, combining ATO with IFNα enhanced and accelerated responses, eradicating MPN in most mice by targeting disease-initiating cells. These results predict potent clinical efficacy of the IFNα+ATO combination in patients and identify PML as a major effector of therapy, even in malignancies with an intact *PML* gene.

## Teaser

Myeloproliferative neoplasms are partly sensitive to interferon α therapy. Dagher et al. demonstrate that arsenic trioxide sharply potentiates interferon’s ability to eliminate patients’ progenitors or disease-initiating cells from mouse models by targeting PML, an interferon target and key senescence gene.

## Graphical Abstract

**Figure:**
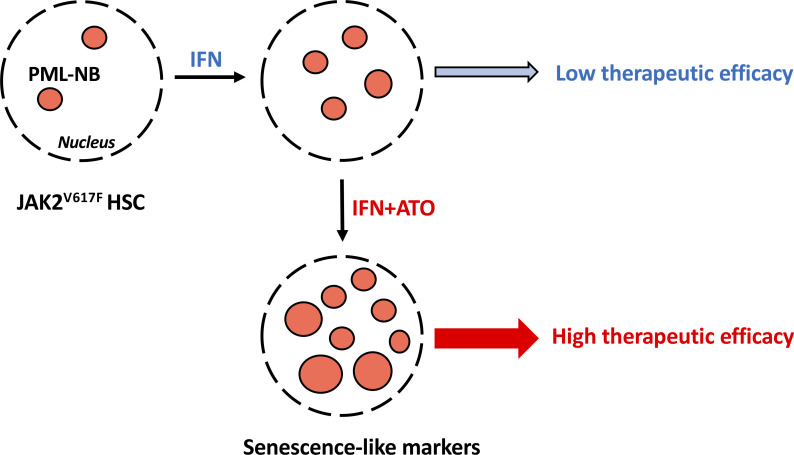

## Introduction

Myeloproliferative neoplasms (MPNs) are chronic stem cell diseases characterized by enhanced production of differentiated blood cells. Most MPN cases are characterized by mutations in *JAK2* that result in the expression of the constitutive active mutant protein kinase JAK2^V617F^ and its downstream cytokine signaling pathways. The *JAK2^V617F^* mutation may yield polycythemia vera (PV), essential thrombocythemia, or primary myelofibrosis (MF), the three diseases that constitute classic *BCR-ABL*–negative MPN. PV, essential thrombocythemia, and MF share common complications, including thrombosis, hemorrhage, and transformation to acute myeloid leukemia (Pasquier et al., 2014; Vainchenker and Kralovics, 2017). Animal models with hematopoietic cells expressing JAK2^V617F^ recapitulate many aspects of the human disease, in particular a PV-like disorder evolving into MF. These animal models have allowed exploration of the biology of JAK2^V617F^ and potential therapeutic approaches (Hasan et al., 2013; Mullally et al., 2013). Apart from phlebotomy in PV, current therapeutic approaches in MPN are based on cytoreductive therapies, JAK2 inhibitors, and IFNα (Spivak, 2019). Although the JAK inhibitor ruxolitinib provides some clinical benefits to MPN patients, it has no significant effect on disease progression and on the JAK2^V617F^ clone. IFNα can target the JAK2^V617F^ clone and yield complete molecular response in 8–20% of patients (Kiladjian et al., 2008; Verger et al., 2018). Yet the molecular mechanisms underlying IFNα therapy remain incompletely understood, precluding improvement of its efficacy (Austin et al., 2020; Hasan et al., 2013; Kiladjian et al., 2016; Mullally et al., 2013).

The PML protein is a member of the TRIM family of ubiquitin/SUMO ligases discovered through its implication in acute promyelocytic leukemia (APL). *PML* is potently transcriptionally activated by IFNs (Stadler et al., 1995). PML has drawn attention from cell biologists due to its ability to nucleate membrane-less organelles, PML nuclear bodies (NBs; Hsu and Kao, 2018; Lallemand-Breitenbach and de Thé, 2018). PML is a sensor for oxidative stress and is essential for induction of senescence (Niwa-Kawakita et al., 2017; Pearson et al., 2000; Vernier et al., 2011). While PML-NBs are disrupted in APL, their reformation drives the therapeutic response (Ablain et al., 2014; Lehmann-Che et al., 2014). Arsenic trioxide (ATO), a curative targeted therapy of APL, directly binds PML to enforce NB formation by promoting PML oxidation and multimerization (Jeanne et al., 2010; Zhang et al., 2010; Zhu et al., 1997). PML-dependent growth arrest and senescence require NB formation (Ivanschitz et al., 2015) so that ATO can enhance some PML activities, notably in IFNα-primed cells (Quignon et al., 1998).

Here, we explored the hypothesis that PML may be a downstream effector of IFNα therapy in MPN. We found that ATO greatly enhances the ability of IFNα to eliminate JAK2^V617F^ MPN stem cells. This elimination is PML dependent and is associated with features of senescence. Our results lay the groundwork for IFNα+ATO-based curative approaches of MPN.

## Results and discussion

### IFNα or ATO enhances PML-NB formation preferentially in JAK2^V617F^ cells

**Figure 1.**
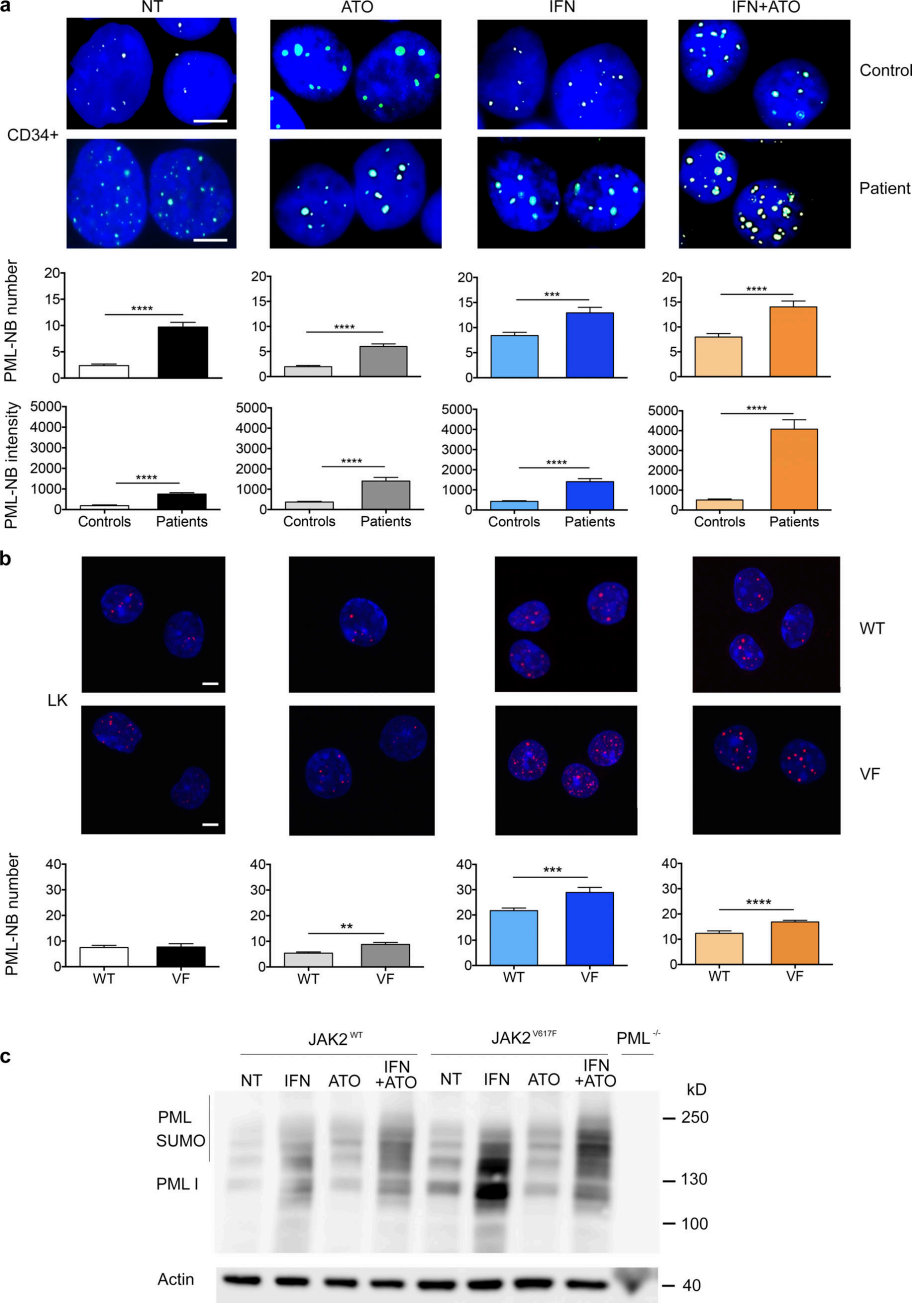
**IFNα+ATO enforces potent NB formation in JAK2^V617F^ cells.** **(a)** Representative immunofluorescence images (upper panel) and quantification of PML-NB numbers and intensities (lower panel) in CD34^+^ cells from a healthy donor (control) and a JAK2^V617F^ MF patient, not treated (NT) or treated in vitro with ATO (1 µM) for 1 h, IFNα (1,000 IU/ml) for 24 h, or both (lower panel represents the pooled results of three patients and controls). Two-tailed unpaired Student’s *t* test. Scale bars = 5 µm. **(b)** Same as panel a in LK cells from *Jak2^V617F^* KI mice (VF) or *Jak2^WT^* littermates (WT) not treated or treated in vitro with ATO (0.3 µM) for 1 h, IFNα (1,000 IU/ml) for 22 h, or both (*n* = 6). Scale bars = 10 µm. **(c)** Western blot analysis of PML in LK cells from *Jak2^V617F^* KI, *Jak2^WT^* littermate, or *Pml^−^*^/^*^−^* mice not treated or treated with ATO for 1 h, IFNα for 22 h, or both (*n* = 3). The values shown are mean ± SEM; **P**< 0.01; ***P**< 0.001; ****P**< 0.0001.

**Figure S1.**
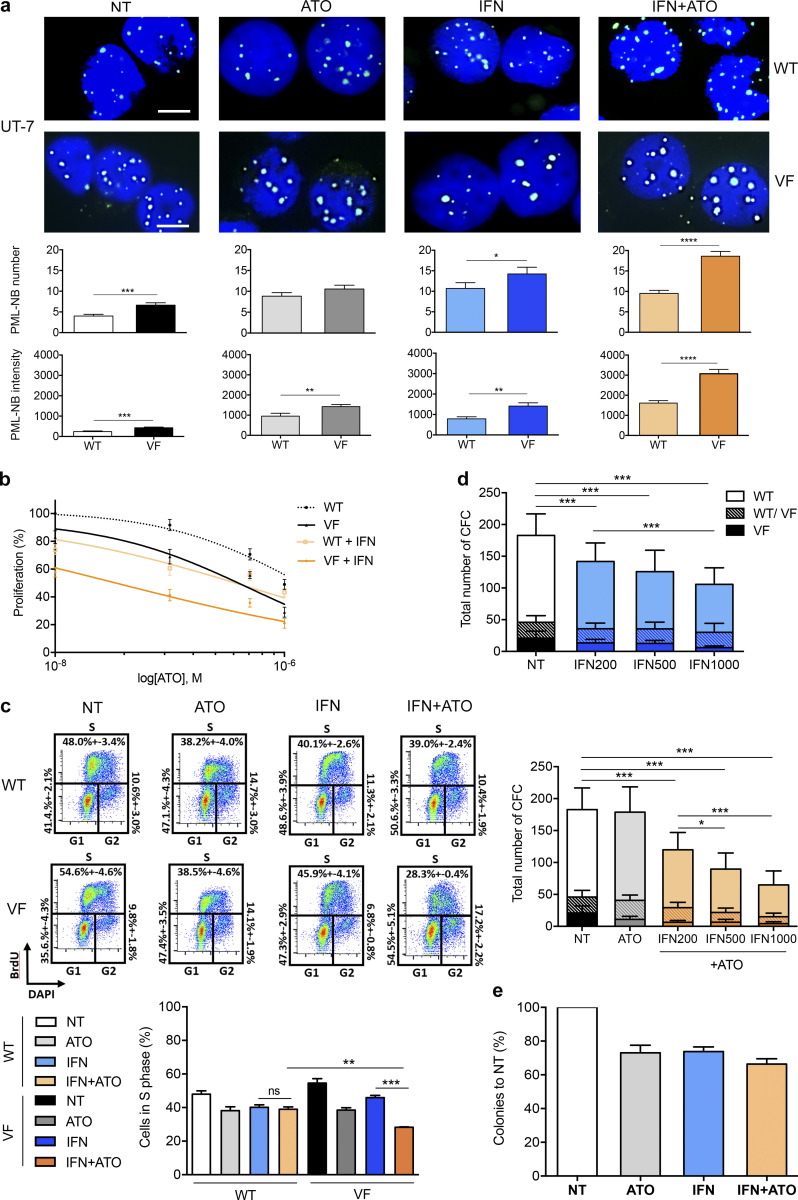
**Arsenic potently enhances NB formation and IFN-driven growth suppression in JAK2^V617F^-expressing cells. (a)** Representative immunofluorescence images (upper panel) and quantification of PML-NB numbers and intensities per cell (lower panel) in JAK2^WT^ (WT) and JAK2^V617F^ (VF) UT-7 cells untreated (NT) or treated with ATO (0.5 µM) for 2 h, IFNα (1,000 IU/ml) for 24 h, or the combination of both drugs in these conditions. Three independent experiments with at least 20 cells analyzed per condition. Two-tailed unpaired Student’s *t* test. Scale bars = 5 µm. **(b)** Proliferation of JAK2^WT^ (WT) or JAK2^V617F^ (VF) UT-7 cells after 72 h of treatment with increasing doses of ATO alone or combined with IFNα 200 IU/ml. Three independent experiments. **(c)** Top panels: example of cell cycle analysis (BrdU/DAPI staining) of either JAK2^WT^ (WT) or JAK2^V617F^ (VF) UT-7 cells 1 d after being untreated or treated with ATO (0.25 µM), IFNα (1,000 IU/ml), or the combination of both. Bottom plot: summary and statistical analysis of three independent experiments. **(d)** Absolute numbers of JAK2^WT/WT^ (WT), JAK2^WT/V617F^ (WT/VF), or JAK2^V617F/V617F^ (VF) colonies counted after 14 d of culture of CD34^+^ cells from seven PV patients. The effect of increasing doses of IFNα (200 IU/ml, 500 IU/ml, and 1,000 IU/ml) is shown on the upper panel, and the effect of increasing doses of IFNα (0 IU/ml, 200 IU/ml, 500 IU/ml, and 1,000 IU/ml) in combination with a constant dose of ATO (0.1 µM) is shown on the lower panel. Seven patient samples in duplicate cultures. Two-tailed unpaired Student’s *t* test. **(e)** Percentages of erythroid colonies relative to untreated controls after 14 d of culture of CD34^+^ cells from five healthy donors untreated or treated with ATO (0.1 µM), IFNα (200 IU/ml), or both drugs in triplicate cultures. Two-tailed unpaired Student’s *t* test. The values shown are mean ± SEM. *P**< 0.05; **P**< 0.01; ***P**< 0.001; ****P**< 0.0001; ns, not significant; CFC, colony-forming cell.

We first explored the PML-NB status of cells expressing JAK2^V617F^. We observed significantly more and larger PML-NBs in CD34^+^ cells from JAK2^V617F^ MF patients compared with control CD34^+^ cells (Fig. 1 a). PML-NB number and staining intensity increased significantly upon IFNα and/or ATO treatment of CD34^+^ cells. A further significant increase of NB staining intensity was found when these cells were treated with both drugs (Lallemand-Breitenbach et al., 2001; Quignon et al., 1998; Zhu et al., 1997; Fig. 1 a). Using human isogenic UT-7 cell lines with or without exogenous JAK2^V617F^, we confirmed that PML-NB formation was consistently more pronounced with JAK2^V617F^ (Fig. S1 a). Mouse bone marrow (BM) Lin^−^c-Kit^+^ (LK) cells harvested from *Jak2^V617F^* knock-in (KI) mice exhibited similar PML-NB numbers as BM LK cells from control littermates. However, after ATO, IFN, or IFN+ATO treatments, *Jak2^V617F^* LK cells consistently contained more PML-NBs than did *Jak2^WT^* LK cells (Fig. 1 b). Western blot analyses performed on these LK cells demonstrated an increased PML level upon ex vivo IFNα treatment (Fig. 1 c). ATO triggered an increase in sumoylated forms of PML accompanied by some decrease in the un-sumoylated PML abundance (Lallemand-Breitenbach et al., 2001, 2008; Fig. 1 c). Importantly, both the basal and IFNα-induced PML levels were consistently higher in LK cells derived from *Jak2^V617F^* KI mice compared with their WT counterparts (Fig. 1 c). Overall, JAK2^V617F^ expression or IFNα exposure increases PML expression, while ATO enforces its NB formation, suggestive of increased sensitivity of mutated cells for PML-controlled processes.

### ATO enhances IFNα-driven growth suppression in JAK2^V617F^ progenitors

**Figure 2.**
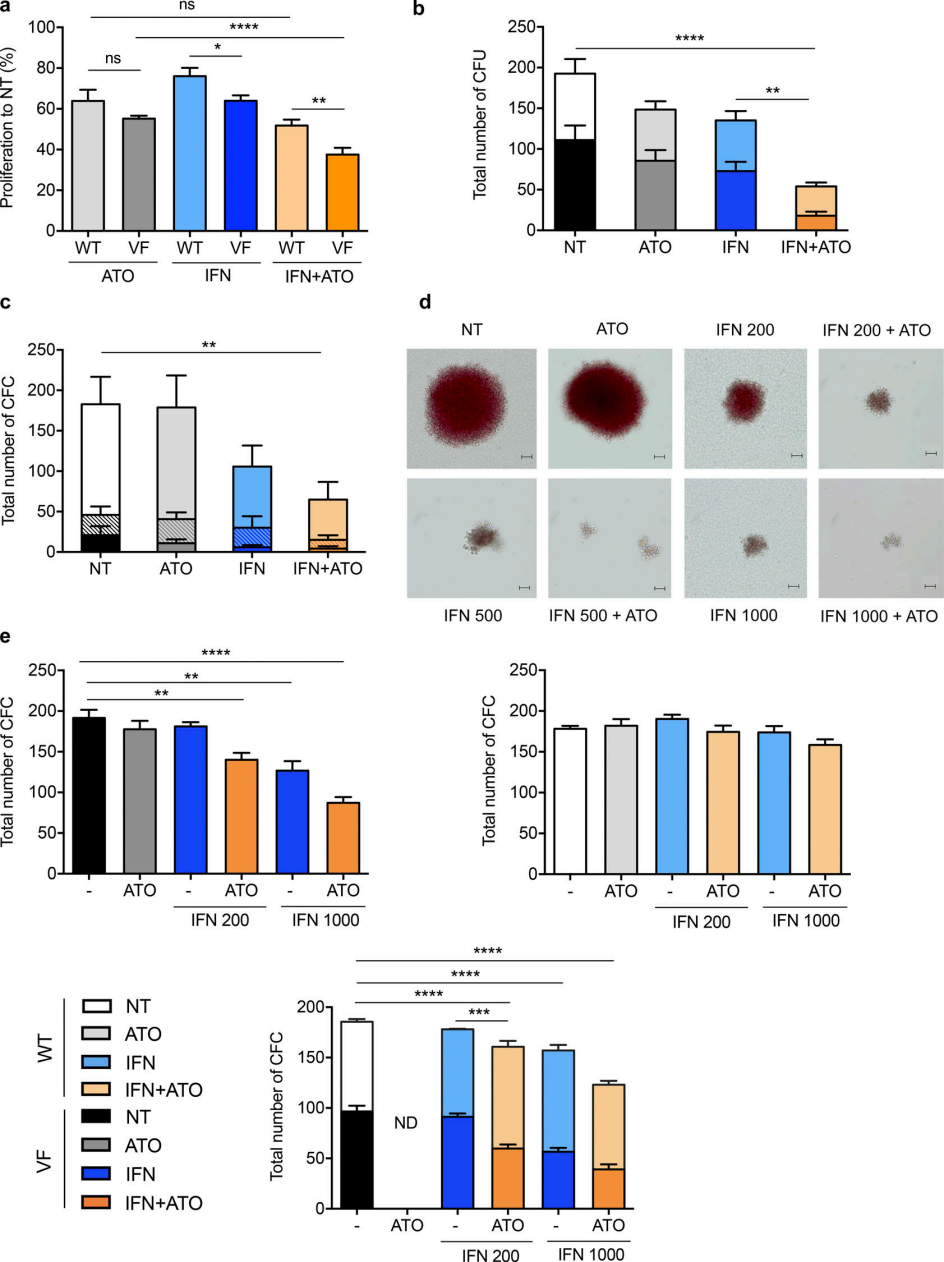
**Arsenic enhances IFNα-driven growth suppression in JAK2^V617F^ cells in vitro.** **(a)** Relative proliferation of *JAK2^WT^* (WT) UT-7 cells or UT-7 cells expressing ectopic JAK2^V617F^ (VF) treated with ATO (0.25 µM), IFNα (1,000 IU/ml), or both for 3 d (*n* = 3). **(b)** Numbers of *JAK2^WT^* (WT, light shade) and *JAK2^V617F^*-positive (VF, dark shade) erythroid colonies from CD34^+^ patient cells cultured for 14 d. Triplicate cultures from 10 *JAK2^V617F^* MF patients untreated (NT) or treated with ATO (0.1 µM), IFNα (200 IU/ml), or both. **(c)** Numbers of *JAK2^WT/WT^* (WT; light shade), *JAK2^WT/V617F^* (WT/VF; patterned), and *JAK2^V617F/V617F^* (VF; dark shade) erythroid and myeloid colonies counted from CD34^+^ patient cells cultured for 14 d. Results represent duplicate cultures from seven PV patients untreated or treated with ATO (0.1 µM), IFNα (1,000 IU/ml), or both. **(d)** Erythroid colonies formed from CD34^+^ cells isolated from a PV patient taken after 14 d of culture: untreated or treated with ATO (0.1 µM), IFNα (200 or 1,000 IU/ml), or both. Scale bars = 50 µm. **(e)** Numbers of erythroid and myeloid colonies after 7 d of culture of 5 × 10^4^ BM cells collected from *Jak2^V617F^*; *Ubi*GFP KI mice (left), from J*ak2^WT^* littermate mice (right), or a 1:1 mixture from both, mimicking the situation found in patients (bottom). Cultures were untreated or treated with ATO (0.1 µM), IFNα (200 or 1,000 IU/ml), or both. Three independent experiments (except for ATO *n* = 1) treated with ATO (0.2 µM), IFNα (200 or 1,000 IU/ml as indicated) with or without ATO. Two-way ANOVA. Error bars represent the mean ± SEM. *P < 0.05, **P < 0.01, ***P < 0.001, and ****P < 0.0001. ns, not significant; CFC, colony-forming cell.

We first investigated the proliferation of UT-7 cells expressing or not exogenous JAK2^V617F^ when treated with increasing concentrations of ATO alone or together with a suboptimal concentration of IFNα (200 IU/ml; Fig. S1 b). Growth inhibition after 3 d was consistently more pronounced in the presence of JAK2^V617F^ (Fig. 2 a). These data were supported by a decreased proportion of cells in S phase, with an enhanced effect between ATO and IFNα (P < 0.001) in UT-7 JAK2^V617F^ cells only (Fig. S1 c).

We then assessed the effect of these drugs on the clonogenic potential of primary cells derived from MF or PV patients, exploring the balance between normal and mutant progenitors by genotyping of individual colonies (Fig. 2, b and c). With cells from the 10 MF patients explored, a decrease in erythroid colony number was observed upon treatment with IFNα, but this effect was considerably enhanced by the IFNα+ATO combination. Importantly, while the proportion of JAK2^V617F^ colonies was not altered by either IFNα or ATO alone, it was significantly decreased (P < 0.01) by the combination (Fig. 2 b). Similarly, in PV patient samples (*n* = 7), IFNα decreased the number of colonies in a dose-dependent manner, and the addition of ATO significantly enhanced this effect (Fig. 2 c and Fig. S1 d). Importantly, the number of JAK2 mutated colonies decreased after treatment with the combination (P = 0.01), but not with IFNα alone (Fig. 2 c). Apart from clonogenic potential, the growth of burst-forming unit–erythroid progenitors was also clearly impacted, as the size of the colonies decreased with IFNα and decreased further with IFNα+ATO (Fig. 2 d). In contrast, IFNα, ATO, and their combination had only moderate effects on the growth of hematopoietic progenitors from healthy individuals or JAK2^WT^ progenitors derived from MF and PV patients (Fig. 2, b and c; and Fig. S1, d and e).

We then explored the anti-clonogenic activity of IFNα and ATO in mouse *Jak2^V617F^* and *Jak2^WT^* hematopoietic progenitors. BM cells from *Jak2^V617F^* KI and *Jak2^WT^* mice were seeded in methylcellulose with cytokines, IFNα, and/or ATO. The *Jak2^V617F^* colony number decreased in response to the IFNα+ATO combination at suboptimal (200 IU) or maximal (1,000 IU) IFNα concentrations, in contrast to *Jak2^WT^* cells (Fig. 2 e). Moreover, the number or proportion of *Jak2* mutant colonies was synergistically decreased by IFNα and ATO, while no single agent had a significant effect. Collectively, ATO significantly enhances the growth-suppressive effects of IFNα on JAK2^V617F^ progenitors ex vivo.

### IFNα+ATO clears JAK2^V617F^ disease-initiating cells in vivo

**Figure S2.**
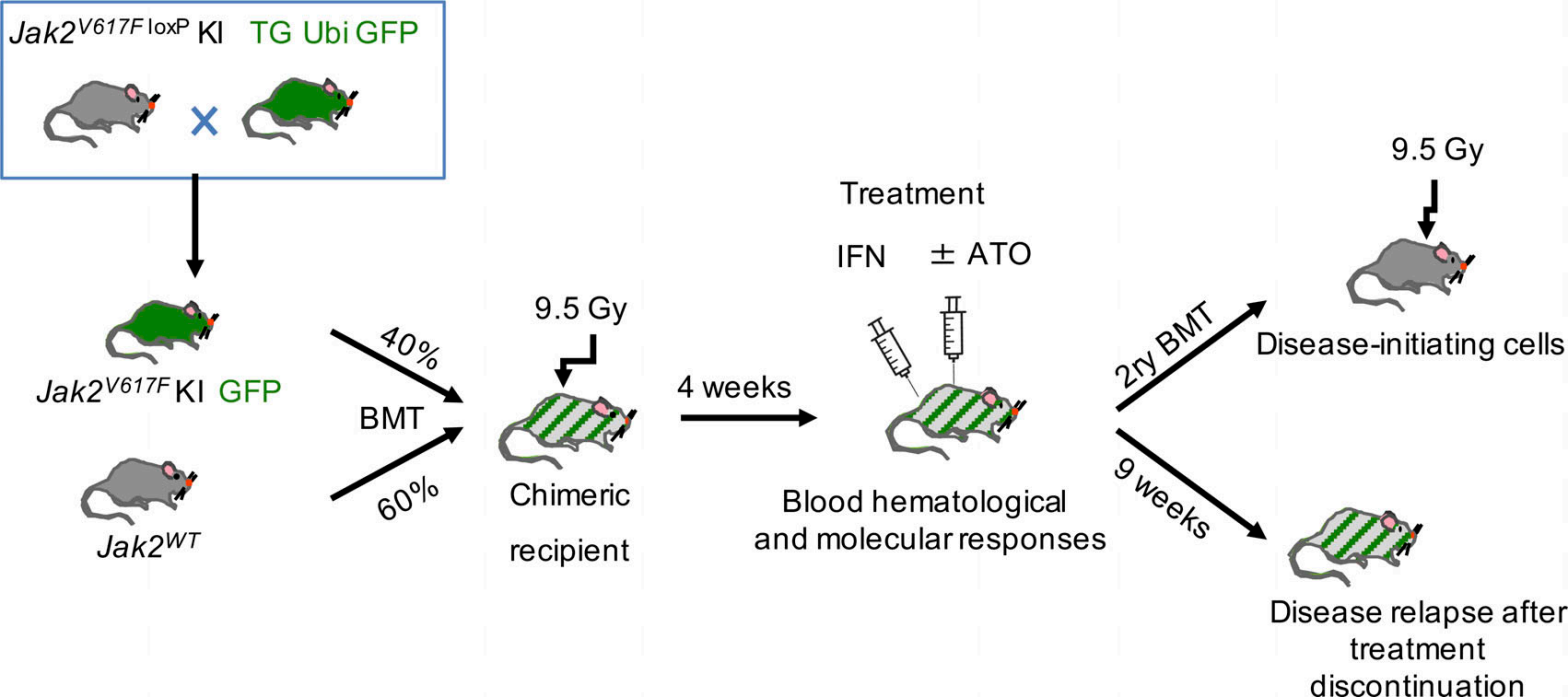
**Experimental design of the preclinical MPN mouse model.** Recipient mice were lethally irradiated and transplanted with 3 × 10^6^ 40:60 mix of *Jak2^V617F^*/*Ubi*GFP and *Jak2^WT^* BM cells. 4 wk after BM transplantation (BMT), mice were treated with ATO (5 mg/kg/2 d), IFNα (3 × 10^4^ IU/d), or both for 8 wk. At completion of treatment, some mice were sacrificed, and their BM cells were used to assess disease-initiating cells in secondary transplantations, also with 3 × 10^6^ BM cells; the remaining mice were kept alive to assess disease relapse around 9 wk without further treatment.

**Figure 3.**
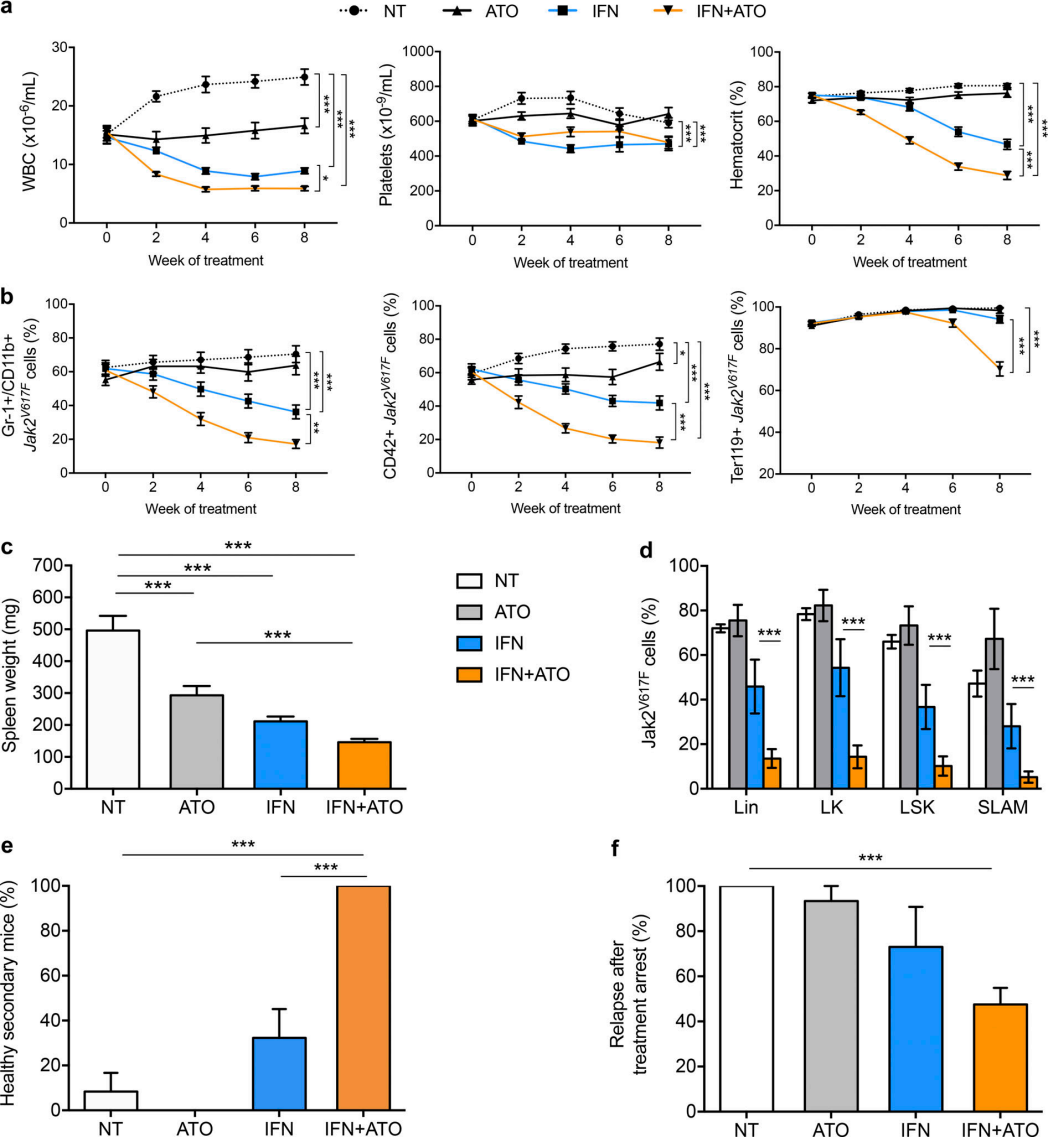
**The IFNα+ATO combination efficiently targets *Jak2^V617F^* disease in vivo.** **(a)** Hematological parameters of mice bearing *Jak2^V617F^* disease were obtained from four independent experiments (untreated, *n* = 25; ATO, *n* = 19; IFNα, *n* = 28; and IFNα+ATO, *n* = 29; Fig. S2 for protocol details). One-way ANOVA with multiple comparisons. **(b)** Percentage of GFP-positive *Jak2^V617F^* Gr-1^+^/CD11b^+^ cells (granulocytes), CD42^+^ cells (platelets), and Ter119^+^ cells (RBCs) in chimeric recipients during treatment. **(c)** Spleen weight after treatment discontinuation obtained from four independent experiments (untreated [NT], *n* = 12; ATO, *n* = 8; IFNα, *n* = 11; and IFN+ATO, *n* = 19). Unpaired two-sided Student’s *t* test. **(d)** Percentage of *Jak2^V617F^* cells (untreated, *n* = 5; ATO, *n* = 4; IFNα, *n* = 4; and IFNα+ATO, *n* = 9). Unpaired two-sided Student’s *t* test. **(e)** Percentage of healthy secondary recipients evaluated at 8–15 wk after BM transplantation (four independent experiments: untreated, *n* = 17; ATO, *n* = 12; IFNα, *n* = 22; and IFNα + ATO, *n* = 18). Mice were considered healthy based on the hematocrit (<50%) and the absence of *Jak2^V617F^* cells (GFP labeled) in granulocytes (Gr-1^+^/CD11b^+^) and platelets (CD42^+^). One-way ANOVA with multiple comparisons. **(f)** Percentage of relapses 9 wk after treatment completion in primary recipients. Results from four independent experiments (untreated, *n* = 11; ATO, *n* = 10; IFNα, *n* = 15; and IFNα + ATO, *n* = 15). One-way ANOVA with multiple comparisons. The values shown are mean ± SEM. *P**< 0.05; **P**< 0.01; ***P**< 0.001.

We next explored the potency of the IFNα+ATO combination in vivo using a *Jak2^V617F^* mouse model (experimental design in Fig. S2), wherein long-term IFNα treatment (14 wk) is required to clear disease-initiating cells in some mice (Hasan et al., 2013). Using an optimized schedule of 5 mg/kg ATO every 2 d with or without IFNα for 8 wk, we found that ATO clearly improved IFNα efficacy to clear MPN (Fig. 3). Although ATO on its own had only modest effects on WBC counts and spleen size, its combination with IFNα significantly accelerated and amplified IFNα-driven hematological response, as assessed by the decrease in WBCs, hematocrit, and splenomegaly (Fig. 3, a and c). Moreover, the addition of ATO significantly increased the molecular response to IFNα, as assessed on blood GFP^+^, *Jak2^V617F^* granulocytes (Gr-1/CD11b), platelets (CD42), and RBC (Ter119) counts (Fig. 3 b). Finally, at the time of sacrifice, combined IFNα+ATO treatment had dramatically decreased the percentage of lineage-negative (Lin^−^), LK, Lin^−^Sca1^+^c-Kit^+^ (LSK), and SLAM^+^ (signaling lymphocyte activation molecule) cells from *Jak2^V617F^* KI origin, demonstrating that the combination is considerably more potent than IFNα alone for targeting *Jak2^V617F^* stem cells (Fig. 3 d).

To substantiate any decline in disease-initiating cells, we transplanted whole BM cells (3 × 10^6^) from treated mice into secondary recipient mice and monitored disease occurrence (Fig. 3 e). Most mice transplanted with BM cells from untreated (*n* = 15/17) or ATO-treated (*n* = 12/12) mice developed the disease, while ∼60% (*n* = 13/22; P = 0.06) of mice transplanted with BM from mice treated with IFNα alone became ill (Fig. 3 e). In contrast, none of the mice transplanted with BM from mice treated with the IFNα+ATO combination (*n* = 0/18; P = 0.0001) developed the disease, strongly suggesting that the combined treatment may have long-term efficacy in primary recipients. We thus monitored disease recurrence in primary animals after treatment discontinuation (Fig. 3 f). All the NT mice remained ill (*n* = 11/11), while 73% of the mice treated with IFNα alone relapsed (*n* = 11/15; P = 0.2) at 9 wk after treatment discontinuation. More than half of the mice (53%) treated with the combination (*n* = 8/15; P = 0.007) remained disease-free 9 wk after treatment discontinuation. Collectively, these results establish that ATO accelerates and amplifies the anti-neoplastic response induced by IFNα, targeting *Jak2^V617F^* stem cells, with long-term clearing of the disease in a substantial number of animals.

### PML drives IFNα+ATO responses

**Figure 4.**
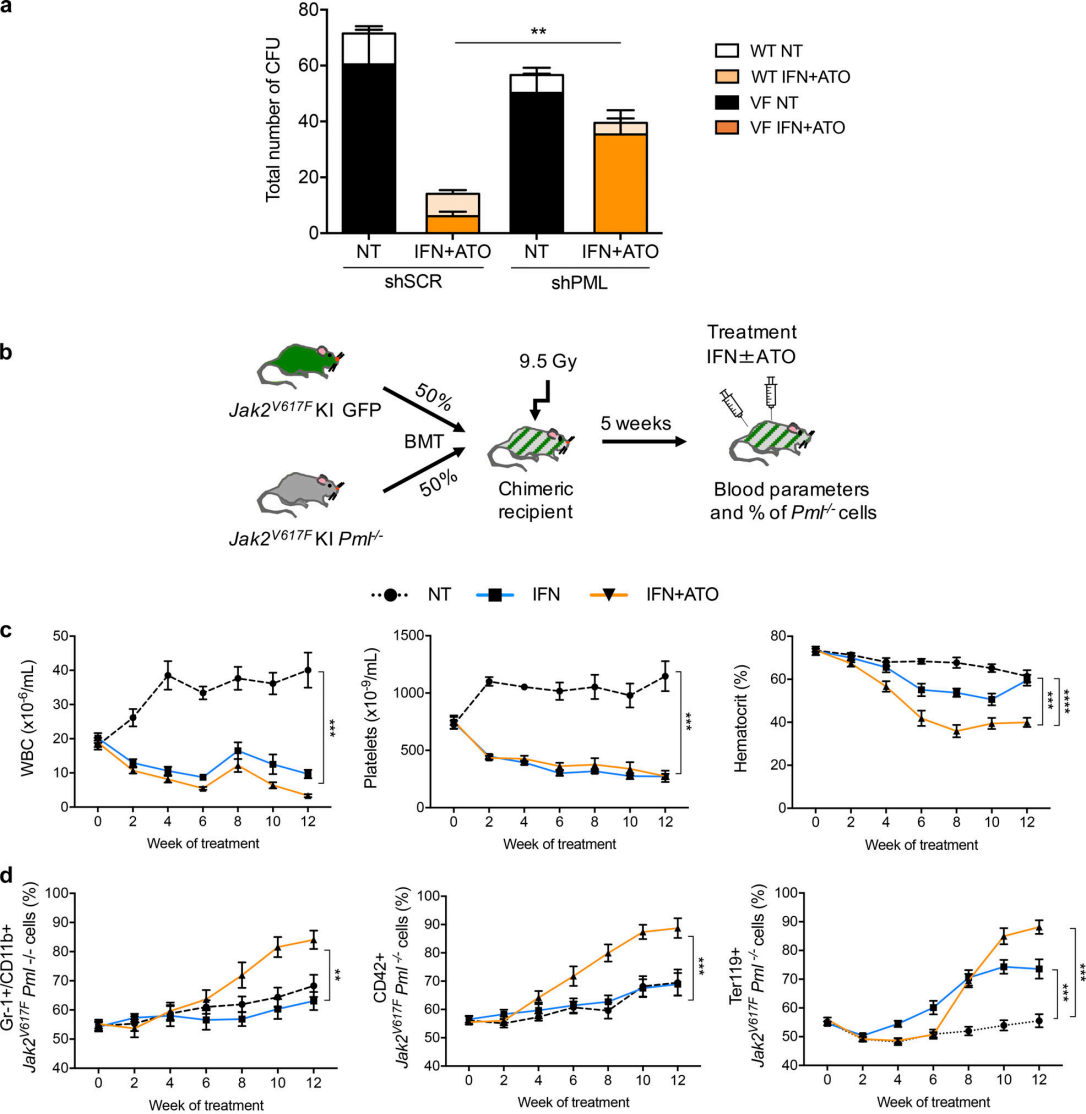
**PML drives IFNα+ATO response.** **(a)** Absolute numbers of *JAK2^WT^* (WT) and *JAK2^V617F^* (VF) erythroid colonies after 14 d of culture of shSCR–treated (PML expressing) or shPML-treated (PML down-regulated) CD34^+^ cells from four JAK2^V617F^ MF patients untreated (NT) or treated with the combination of IFNα (200 IU/ml) and ATO (0.1 µM). Unpaired two-sided Student’s *t* test. **(b)** Lethally irradiated mice were grafted with 50% BM cells from *Jak2^V617F^* KI/*Ubi*GFP mice and 50% BM cells from *Jak2^V617F^* KI *Pml*^−/−^ mice. Mice were treated with IFNα alone or IFNα+ATO 4 wk after transplantation. **(c)** Blood parameters (WBCs, platelets, and hematocrit) every 2 wk during the treatment. *n* = 5 for each treatment; one-way ANOVA with multiple comparisons. **(d)** Percentage of *Pml*^−/−^ Gr-1^+^ cells (granulocytes), *Pml*^−/−^ CD42^+^ cells (platelets), and *Pml*^−/−^ Ter119^+^ cells (RBCs). *n* = 5 for each treatment; one-way ANOVA with multiple comparisons. Color code as in panel c. The values shown are mean ± SEM. **P**< 0.01; ***P**< 0.001; ****P**< 0.0001. BMT, BM transplantation.

The IFNα+ATO combination maximizes PML-NB formation (Quignon et al., 1998). To explore any role of PML in the action of the IFNα+ATO combination, we first examined the role of PML knockdown in CD34^+^ cells from *JAK2^V617F^* MF patients. After transduction with lentiviruses expressing an shRNA efficiently targeting PML (Ivanschitz et al., 2015; Niwa-Kawakita et al., 2017), CD34^+^ cells were sorted for GFP expression, seeded in semisolid cultures, and treated or not with IFNα and/or ATO. Remarkably, the decrease in mutant colony formation upon IFNα+ATO treatment was severely blunted by PML down-regulation (Fig. 4 a). These results strongly suggest that the ability of IFNα+ATO to target the clonogenic activity of JAK2^V617F^ progenitors is PML dependent.

To further explore PML contributions to IFNα or/and ATO selective elimination of JAK2^V617F^ hematopoietic stem cells (HSCs) in vivo, we generated *Jak2^V617F^*/*Pml*^−/−^ mice and transplanted syngenic mice with a mixture of *Jak2^V617F^*/*Pml*^−/−^ and *Jak2^V617F^*/*Pml*^+/+^ BM cells (Fig. 4 b). 5 wk after transplantation, mice were treated with IFNα and/or ATO for 12 wk. In this experimental setup, if *Pml* was required for therapy response, its absence should yield a progressive survival advantage to *Jak2^V617F^*/*Pml*^−/−^ cells. In NT animals, MPN rapidly developed (Fig. 4 c). The IFNα+ATO combination or IFNα alone elicited a rapid decrease of leukocytosis, thrombocytosis, and erythrocytosis (Fig. 4 c). A time-dependent enrichment of *Jak2^V617F^*/*Pml*^−/−^ granulocytes, platelets, and RBCs occurred in response to combined IFNα+ATO therapy (Fig. 4 d), while this enrichment was observed for IFNα alone only in *Jak2^V617F^*/*Pml*^−/−^ RBCs. This key observation demonstrates *Pml-*dependent targeting of HSCs by IFNα+ATO in vivo and a partial *Pml* dependency for IFN response. Collectively, these results demonstrate that PML is involved in the elimination of JAK2^V617F^ progenitors or stem cells in response to the IFNα+ATO combination.

### IFNα+ATO drives PML-dependent senescence ex vivo

**Figure S3.**
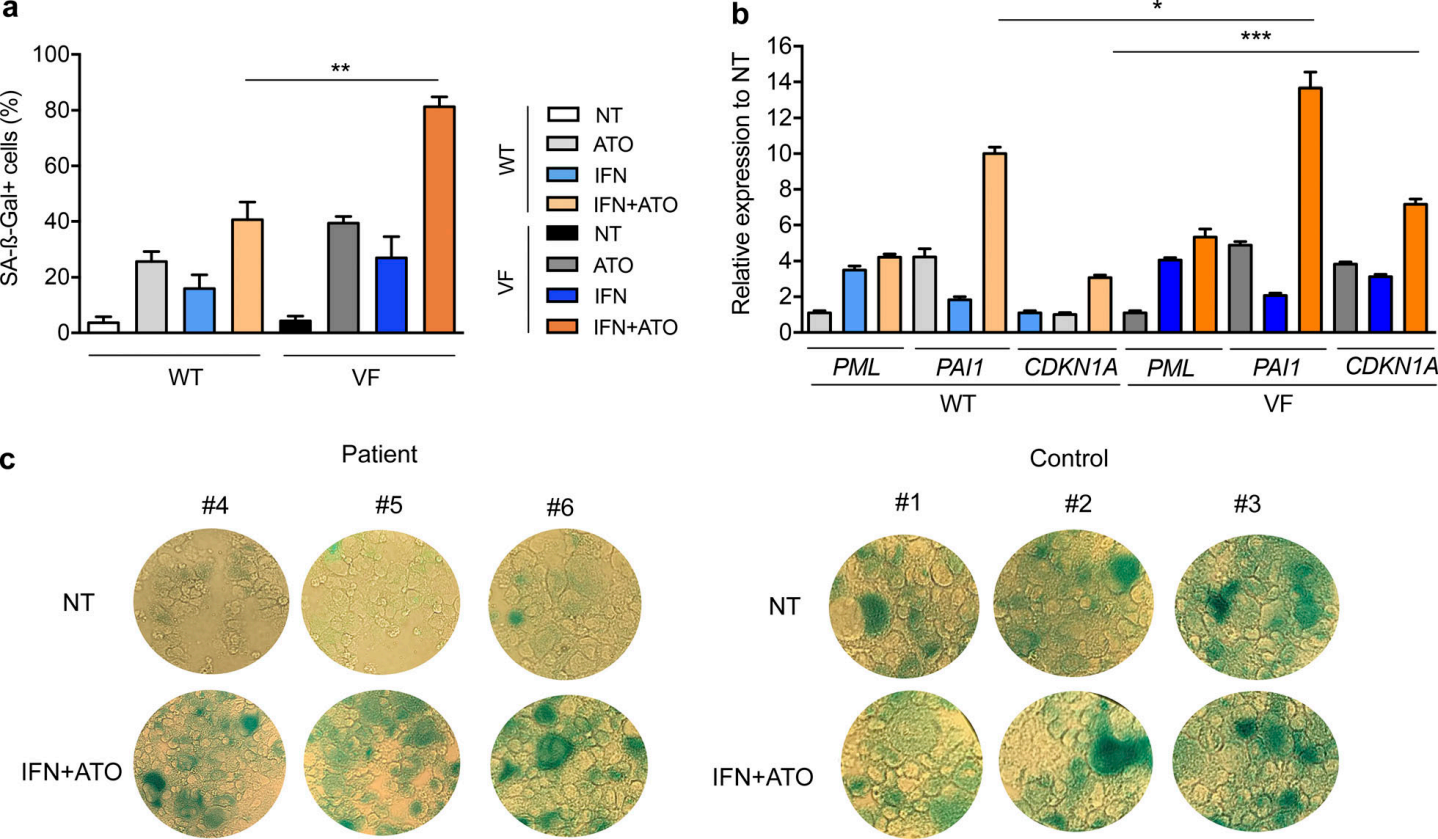
**IFN/ATO drives senescence more efficiently in the JAK2^V617F^ context.** **(a)** Percentages of JAK2^WT^ (WT) or JAK2^V617F^ (VF) UT-7 cells untreated (NT) or treated with ATO (0.25 µM), IFNα (1,000 IU/ml), or both drugs for 7 d stained positively for SA-β-Gal. **(b)** Relative expression to untreated control of the senescence-associated genes *PML*, *PAI1*, and *CDKN1A* in UT-7 cells. Color code as in panel a. Error bars represent mean ± SEM of three independent experiments; unpaired two-sided Student’s *t* test. **(c)** SA-β-Gal staining assay of CD34^+^-derived cells from three MF patients or three healthy controls after 10 d of megakaryocytic differentiation and untreated or treated with the combination of IFNα (1,000 IU/ml) and ATO (0.25 µM). The values shown are mean ± SEM. *P**< 0.05; **P**< 0.01; ***P**< 0.001.

**Figure 5.**
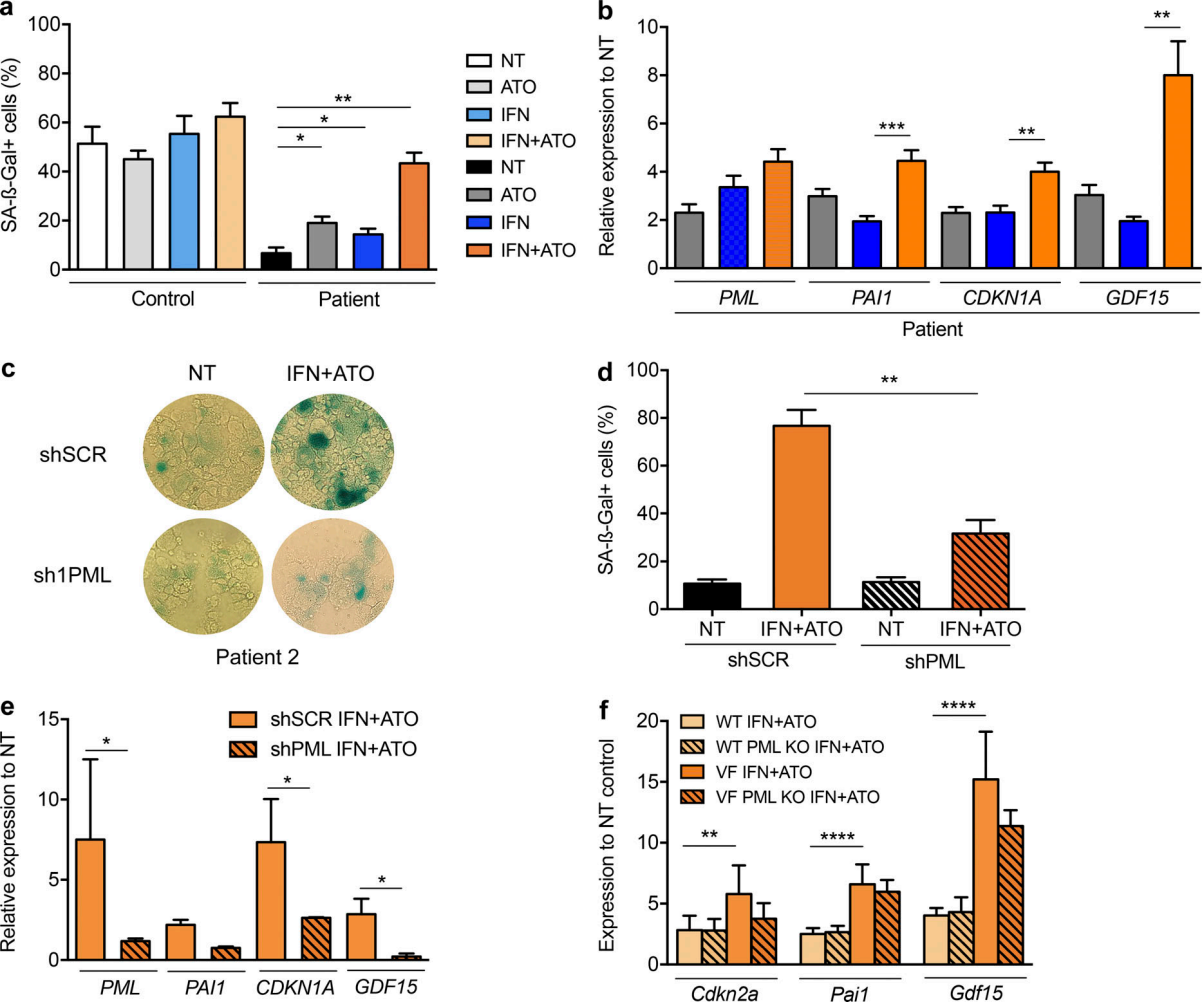
**IFNα+ATO drives PML-dependent senescence.** **(a)** Percentages of SA-β-Gal^+^ cells after CD34^+^ cells from three healthy donors’ (control) and three *JAK2^V617F^* MF patients’ samples (patient) were treated with ATO (0.25 µM), IFNα (1,000 IU/ml), or both for 10 d. Unpaired two-sided Student’s *t* test. **(b)** Relative expression to untreated control of senescence-associated genes *PML*, *PAI1*,* CDKN1A*, and *GDF15* of patient samples described in panel a. Color code as in panel a. Unpaired two-sided Student’s *t* test. **(c)** SA**-**β-Gal staining of shSCR–treated (PML expressing) or shPML-treated (PML down-regulated) CD34^+^-derived cells from two MF patients after 10 d of megakaryocytic differentiation and untreated (NT) or treated with the combination IFNα (1,000 IU/ml) and ATO (0.25 µM). **(d)** Percentages of SA-β-Gal^+^ cells of shSCR–treated (PML-expressing) or shPML-treated (PML down-regulated) CD34^+^-derived cells from three MF patient samples after 10 d of megakaryocytic differentiation untreated or treated with the combination IFNα (1,000 IU/ml) and ATO (0.25 µM). **(e)** Relative expression of *PML*, *PAI1, CDKN1A*, and *GDF15* of samples described in panel d. Unpaired two-sided Student’s *t* test. **(f)** Expression of *Cdkn2a*, *Pai1,* and *Gdf15* in mouse Lin^−^ BM cells derived from *Jak2^V617F^* KI (VF) or littermate *Jak2^WT^* (WT) mice deficient (hatched bars) or not (not hatched bars) in *Pml* treated in vitro with IFNα (1,000 IU/ml)+ATO (0.3 µM) for 24 h. The values shown are mean ± SEM. *P**< 0.05; **P**< 0.01; ***P**< 0.001; ****P**< 0.0001.

In the syngenic UT-7 model, IFNα and/or ATO induced SA-β–galactosidase activity and expression of senescence-associated genes, which were significantly enhanced in JAK2^V617F^*-*expressing cells (Fig. S3, a and b). We then compared the behavior of CD34^+^ hematopoietic progenitors from healthy individuals and MPN patients in a condition of megakaryocytic differentiation. Normal cells exhibited massive spontaneous senescence, while those derived from MF patients did not (Besancenot et al., 2010). Yet IFNα+ATO treatment restored senescence in MF patient cells, as assessed by SA-β–galactosidase activity and expression of senescence-associated genes (Fig. 5, a and b; and Fig. S3 c). Induction of senescence by the IFNα+ATO combination in primary cultures of progenitors from JAK2^V617F^ MF patients was abrogated upon lentiviral-introduced shRNA targeting PML (Fig. 5, c–e).

We similarly explored ex vivo IFNα and ATO treatments of primary BM progenitor cells derived from *Jak2^V617F^* KI or littermate *Jak2^WT^* mice, deficient or not for *Pml*. The expression of senescence-associated genes (*Cdkn2a*, *Pai1*, and *Gdf15*) was significantly more induced by the IFNα+ATO combination in *Jak2^V617F^* Lin^−^ cells than in *Jak2^WT^* Lin^−^ cells, and the absence of *Pml* blunted their induction (Fig. 5 f). Collectively, the IFNα+ATO combination preferentially induces senescence markers in JAK2^V617F^ primary cells, at least in part through PML.

Although IFNα targets JAK2^V617F^ hematopoietic progenitors, in patient or mouse models its long-term efficacy remains modest (Austin et al., 2020; Gisslinger et al., 2020; Hasan et al., 2013; Kiladjian et al., 2006; Mullally et al., 2012). Several clinical and preclinical studies have attempted to improve IFNα efficacy, for example, through combinations with MDM2 or JAK2 inhibitors (Austin et al., 2020; Lu et al., 2014; Mascarenhas et al., 2019). We demonstrate that the IFNα+ATO combination specifically targets the *Jak2^V617F^* disease-initiating cells in vivo, with a much higher potency than IFNα alone. These cooperative effects were observed in a wide variety of JAK2^V617F^-expressing models, including primary hematopoietic progenitors, suggestive of therapeutic relevance. In our setting, IFNα has cell-autonomous activity, clearly independent from its action on anti-tumor immunity (Zitvogel et al., 2015). We demonstrate that improvement of IFNα efficacy by ATO primarily relies on PML and provide some evidence for a contribution of senescence. PML-NBs promote p53 activation and E2F shutoff (Vernier and Ferbeyre, 2014), opposing tumor development in multiple settings (Salomoni and Pandolfi, 2002). PML-NBs may also control apoptosis (Giorgi et al., 2010; Takahashi et al., 2004). Mechanistically, these growth-suppressive effects could rely on promotion of post-translational modifications—notably sumoylation—by PML-NBs (Sahin et al., 2014; Seeler and Dejean, 2017). That *Jak2^V617F^* HSCs exhibit high basal levels of PML and reactive oxygen species (Marty et al., 2013) may explain their higher sensitivity to NB biogenesis enforced by the IFNα+ATO combination. Our findings highlight an unexpected parallel with APL, where PML-NB reformation is required for eradication of the disease (Ablain et al., 2014; de Thé et al., 2017; Lehmann-Che et al., 2014). PML may thus represent a common effector pathway in cancer types where its gene is not rearranged. Clinically, our studies predict that ATO administration should greatly increase IFNα-driven responses in MPN patients. The IFNα+ATO combination had a favorable safety profile in another hematologic malignancy (Kchour et al., 2009), and availability of oral forms of ATO (Zhu et al., 2018) may allow outpatient therapy, paving the way toward eradication of JAK2^V617F^ MPN cells.

## Methods and materials

### Animal experiments

The conditional flexed *Jak2^V617F^* KI mice were described (Hasan et al., 2013). To express the mutant *Jak2*, KI mice were crossed with *Vav*Cre transgenic (TG) mice (Croker et al., 2004). To easily evaluate the allele burden, we crossed *Jak2^V617F^* KI/*Vav*Cre mice with *Ubi*GFP TG mice (Schaefer et al., 2001). To explore the PML contribution to IFN response, we first generated C57BL/6 mice with a germinal deletion of Pml exon 3 by crossing *Pml^f/f^* mice (Amodeo et al., 2017) with *CMV*-Cre TG mice. Excision was confirmed by PCR and Western blot. These were crossed with C57BL/6 *Jak2^V617F^* KI/*Vav*Cre mice or *Ubi*GFP TG mice. For primary or secondary BM transplantation, 3 × 10^6^ BM cells were engrafted into lethally irradiated (9.5 Gy) congenic recipient mice. To reproduce patient features, where normal hematopoiesis persists with the neoplastic clone (s), the preclinical mouse models were generated from normal recipient mice transplanted with a mixture of 40% BM cells collected from *Jak2^V617F^* KI/*Vav*Cre/*Ubi*GFP mice, and 60% BM cells were collected from WT C57BL/6 mice. Murine IFNα (Miltenyi Biotech; 5 × 10^7^ IU/ml) was subcutaneously injected every day at doses of 3 × 10^4^ IU in 0.1 ml PBS/mouse (1.5 × 10^6^ IU/kg). ATO (As_2_O_3_, product number A1010; Sigma-Aldrich) was injected intraperitoneally at 5 mg/kg every 2 d.

Hemoglobin, mean corpuscular volume, hematocrit, RBC counts, platelet, and WBC counts were determined using an automated counter (MS9; Melet Schloesing Laboratoires) on blood collected from the retro-orbital plexus in EDTA tubes. BM cells were collected by flushing both femurs and tibias. Spleens were weighed, and single-cell suspensions were prepared. Relapse after therapy was evaluated based on the hematocrit (<50%), the allele burden (GFP^+^) in granulocytes (Gr-1^+^/CD11b^+^) or RBCs (Ter119^+^), and the spleen weight (<150 mg).

### Flow cytometry

Flow cytometry of mouse blood, BM, and spleen was used to determine the *Jak2^V617F^* allele burden as the percentage of GFP-positive cells in tissues (CANTO X, DIVA6 analyzer; Becton Dickinson). A small sample of blood was labeled with APC anti–TER-119 antibody (Ab; clone TER-119; BioLegend). The rest of the blood sample, after RBC lysis, was incubated with purified anti-CD16/32 Ab (clone 93; BioLegend) for 10 min at room temperature and then divided into two samples. The first one was labeled with APC-conjugated anti-CD42d Ab (clone 1C2; BioLegend) for platelets. The second one was labeled with PE/Cy7-conjugated anti–Ly-6G/Ly-6C (Gr-1) Ab (clone RB6-8C5; BioLegend) and PerCP/Cyanine5.5-conjugated anti-CD11b Ab (clone M1/70; BioLegend) for granulocytes, PE-conjugated anti-CD45R/B220 Ab (clone RA3-6B2; BioLegend) for lymphocytes B, and APC-conjugated anti-CD3 Ab (clone 17A2; BioLegend) for lymphocytes T. For the BM sample, Lin^−^ cells were enriched by immunomagnetic negative selection (IMag; BD Biosciences) and then sorted or assessed by flow cytometry using APC-conjugated lineage panel Abs (anti–TER-119 [clone TER-119], anti–Ly-6G/Ly-6C [Gr-1] Ab [clone RB6-8C5], anti-CD11b Ab [clone M1/70], anti–CD45R/B220 Ab [clone RA3-6B2], and anti-CD3 Ab [clone 17A2]). LK cells were analyzed from Lin^−^ cells labeled with PerCP/Cyanine5.5–conjugated or PE-conjugated anti-CD117 Ab (c-Kit; clone 2B8; BioLegend). LSK cells were analyzed from Lin^−^ cells labeled with PerCP/Cyanine5.5-conjugated or PE-conjugated anti-CD117 Ab (c-Kit; clone 2B8) and PE/Cy7-conjugated anti–Ly-6A/E Ab (Sca-1; clone D7; BioLegend). SLAM marker fractionated cells were analyzed from LSK cells labeled with APC/Cyanine7-conjugated anti-CD48 Ab (clone HM48-1; BioLegend) and Brilliant Violet 421–conjugated anti-CD150 Ab (clone TC15-12F12.2; BioLegend). After in vitro culture, viable cells were sorted after staining with SYTOX Blue Dead Cell Stain (S34857; Invitrogen by Thermo Fisher Scientific). In human cell lines, the cell cycle distribution was measured via incorporation of BrdU (Phase-Flow; 370706; BioLegend) and DAPI **(**Thermo Fisher Scientific) labeling by flow cytometry on a FACS Canto II (Becton Dickinson) following the manufacturer’s recommendations at day 1 after treatment with ATO, IFN, or both.

### Immunofluorescence microscopy

Human cell lines, patient-derived cells, or mouse Lin^−^ or LK cells were treated with ATO alone, IFN alone, or both and then washed with PBS, cytospined, fixed using 4% paraformaldehyde (Thermo Fisher Scientific) for 10 min, and permeabilized with 0.5% (human) or 0.2% (mouse) Triton X-100. Human cells were labeled with a murine monoclonal Ab detecting human PML and a secondary anti-mouse Ab conjugated to Alexa Fluor 488 (Thermo Fisher Scientific; Lallemand-Breitenbach et al., 2001). Mouse monoclonal anti-mouse PML, clone 36.1–104 (MAB3738; Merck Millipore) was directly coupled to FITC fluorochrome or Alexa Fluor A594 for immunostaining. Slides were washed and then incubated in DAPI 1 µg/ml. After several washes, the slides were mounted in Vectashield H-1000 Mounting medium (Vector Laboratories, Inc.). For human samples, slides were kept at +4°C and analyzed under confocal microscope (LSM 800; Zeiss) under a 63× objective oil immersion. Z-stack images were processed with the image software ZEN (Zen 2 blue edition, 2011; Carl Zeiss Microscopy GmbH), and dots were counted and intensity measured on at least 20 cells spread over five to six fields with the image analysis software ImageJ (ImageJ2, Dec 2009). Mouse samples were imaged with a reversed confocal point-scanning microscope (SP8-Leica Microsystem) equipped with four lasers, 405, 488, 561, and 638 nm. An oil immersion, high numerical aperture (1.40) 63× objective was used for imaging. The scan protocol was preserved throughout all experiments. Two sequences were used to spectrally separate the two wavelengths, one for DAPI and the other for Alexa Fluor 594. The emission signal was collected on two HyD detectors for higher sensitivity. For the 594-nm signal, the “photon-counting” mode was used to remove any false numerical gain of signal. At least 20 cells per condition were analyzed using an ImageJ plug-in developed by Collège de France microscopy core facilities.

### Cell lines and primary cells

The human megakaryoblastic UT-7 11oc1 cell line was a kind gift of Dr. Komatsu (Juntendo University Graduate School of Medicine, Tokyo, Japan). These cells were lentivirally transduced either by JAK2^WT^- or JAK2^V617F^-expressing vectors as previously described (Besancenot et al., 2010). They were grown in DMEM-10% FBS-1% penicillin-streptomycin in the presence of erythropoietin (1 IU/ml). Human erythroleukemia cells (obtained from the American Type Culture Collection) were grown in RPMI 1640 medium supplemented with 10% FBS and antibiotics (100 mg/ml penicillin-streptomycin mix; Thermo Fisher Scientific). Human CD34^+^ cells were purified from peripheral blood mononuclear cells of PV/MF patients using the human CD34 MicroBead Kit from Miltenyi Biotec. Peripheral blood mononuclear cells from healthy donors were obtained at the French National Blood Service (French transfusion institution).

Human CD34^+^ cells were cultured in StemSpan medium (StemCell Technologies, Inc.) in the presence of recombinant human cytokines (PeproTech France): thrombopoietin (20 ng/ml), stem cell factor (SCF; 100 ng/ml), and IL-3 (50 ng/ml) for 10 d for megakaryocytic differentiation. Cultured cells were maintained in a humidified atmosphere at 37°C with 5% CO_2_. Cell lines were determined to be mycoplasma free. For human studies, we used IFNα-2a (Roferon-A; Roche or Merck Millipore) and ATO (As_2_O_3_, 01969, or A1010).

Mouse BM-derived Lin^−^ cells were cultured in serum-free MSS medium (Iscove modified Dulbecco’s medium with penicillin/streptomycin/glutamine, α-thioglycerol, bovine serum albumin, sonicated lipids, and insulin-transferrin; Amgen) supplemented with cytokines: murine SCF (50 ng/ml), murine IL-11 (100 ng/ml), and human FLt3 (50 ng/ml; Lui et al., 2014). Cultured cells were maintained in a humidified atmosphere at 37°C with 5% CO_2_. Cells were treated with ATO (0.1 µM, 0.2 µM, or 0.3 µM), IFNα (200 IU/ml, 500 IU/ml, or 1,000 IU/ml; Miltenyi Biotech; 5 × 10^7^ IU/ml), or both drugs.

### CFU assays

Human clonogenic assays were performed by seeding in duplicates or triplicates 1,500–5,000 CD34^+^ cells in MethoCult H4434 with cytokines (StemCell Technologies, Inc.) or MethoCult H4230 (StemCell Technologies, Inc.) with the following mixtures of cytokines: 25 ng/ml SCF (Biovitrum AB), 10 ng/ml IL-3 (Miltenyi Biotech), and 1 U/ml erythropoietin. Cultures were treated with ATO (0.1 µM), or IFNα (200–1,000 IU/ml), or both drugs. After 14 d of incubation, Burst-forming unit–erythroid, CFU-granulocyte, CFU-macrophage, and CFU-granulocyte-macrophage colonies were enumerated. Then, at least 40 colonies per condition were genotyped for *JAK2*^*V617F*^ with a TaqMan single-nucleotide polymorphism genotyping assay using the 7500 Real Time PCR System (Applied Biosystems). We used forward (5′-AAG​CTT​TCT​CAC​AAG​CAT​TTG​GTT​T-3′) and reverse (5′-AGG​CAT​TAG​AAA​GCC​TGT​AGT​TTT​ACT​T-3′) primers and allele-specific probes (WT: 5′-HEX-TGTGTCTGTGGAG-TAMRA-3′; V617F: 5′-6-FAM-TGTTTCTGTGGAG-TAMRA-3′).

Mouse BM cells from *Jak2^V617F^* KI or *Jak2^WT^* littermate mice were used pure or mixed at 50% ratio and treated or not with IFNα 200 and 1,000 IU/ml (Miltenyi Biotech) with or without ATO 0.2 µM (As_2_O_3_, A1010). Cells were seeded at a density of 5 × 10^4^/ml in semi-solid cultures (Methocult, M3434; Stem Cell Technologies) supplemented with thrombopoietin (10 ng/ml). Triplicate experiments were performed, twice with BM cells from *Jak2^WT^* and *Jak2^V617F^* GFP^+^ and once with *Jak2^WT^* GFP^+^ and *Jak2^V617F^*.

### Viral transductions

CD34^+^ cells derived from MPN patients were transduced by a pLVTHM-eGFP–shPML lentiviral vector (Ivanschitz et al., 2015; Niwa-Kawakita et al., 2017). Briefly, lentiviral vectors were packaged in 293T cells by transfecting pCMV-8.91 and pMD.G (Addgene) for 2 d. The supernatants containing the lentiviruses were concentrated with RetroX solution (Takara Bio Europe). Hematopoietic cells were then spinoculated for 90 min with the lentiviral supernatant and then cultured for 2 d. On the third day, the lentiviral supernatant was removed, and the percentage of transduced cells was evaluated by the percentage of GFP-positive cells in a FACS Canto II and then sorted in a FACS Aria III (Becton Dickinson).

### Western blot

Cell pellets were lysed in 2× Laemmli buffer, sonicated on ice for 15 min, and denatured for 7 min at 95°C. Samples were separated by a 4–12% polyacrylamide gel. Proteins were transferred on a nitrocellulose membrane for 2 h. The membrane was blocked with 10% milk–Tris-buffered saline–Tween 0.1% and then incubated overnight at 4°C with mouse monoclonal anti-mouse PML, clone 36.1–104 (MAB3738). After several washes, the membrane was incubated with anti-mouse secondary Ab (Jackson ImmunoResearch) for 1 h at room temperature and washed, and protein expression was revealed using a luminescent Image Analyzer (ImageQuant LAS 4000; GE Healthcare Bio-Sciences).

### Cell analysis

For proliferation assays, cells were seeded in triplicates at 3 × 10^5^/ml in a 96-well plate in the presence of IFN, ATO, or both. Cells were counted at indicated time points with a TC20 counting apparatus (Bio-Rad), and cell viability was measured using trypan blue. Senescence-associated β-galactosidase (SA-β-Gal) staining was performed in vitro on cells at 10 d of treatment with a Senescence β-Galactosidase Staining Kit (9860; Cell Signaling) following the manufacturer’s recommendations. The percentages of stained cells were counted in 20 fields in each condition using an optical microscope in >4,000 cells.

### Quantitative PCR of gene transcripts

Total RNA was isolated from cell lines or cells derived from human primary CD34^+^ cells after 10 d of culture. RNA was extracted using Trizol (Thermo Fisher Scientific). First-strand cDNA was synthesized using SuperScript III reverse transcription (Thermo Fisher Scientific). Quantitative RT-PCR was performed using TaqMan Fast Universal PCR Master Mix on a 7500 Fast Real-Time PCR System (Applied Biosystems). Predesigned TaqMan assays for *PML*, *CDKN1A*, *PAI1*, and *GDF15* genes were purchased from Applied Biosystems. *TBP* was used as an endogenous control to calibrate the amount of target mRNA. Mouse Lin^−^ cells were cultured during 24 h, and then total RNA was extracted using Direct-zol RNA MicroPrep (Zymo Research) and cDNA was synthesized using SuperScript IV VILO Master Mix with the ezDNase Enzyme kit (Thermo Fisher Scientific). Quantitative RT-PCR was performed in an Applied Biosystems 7500 Real-Time PCR System thermocycler using the TB Green Premix Ex Taq (Takara Bio Europe). mRNA was normalized by measuring HPRT mRNA levels. The following primers were used: *Cdkn2a* forward 5′-GCT​CTG​GCT​TTC​GTG​AAC​AT-3′ and *Cdkn2a* reverse 5′-TAC​GTG​AAC​GTT​GCC​CAT​CA-3′; *Pai1* forward 5′-TGG​GTG​GAA​AGG​CAT​ACC​AAA-3′ and *Pai1* reverse 5′-AAG​TAG​AGG​GCA​TTC​ACC​AGC-3′; *Gdf15* forward 5′-GAG​CTA​CGG​GGT​CGC​TTC-3′ and *Gdf15* reverse 5′-GGG​ACC​CCA​ATC​TCA​CCT-3′; and *Hprt* forward 5′-AAG​ACT​TGC​TCG​AGA​TGT​CAT​GAA-3′ and *Hprt* reverse 5′-ATC​CAG​CAG​GTC​CCT​TGA​ACC​TT-3′.

### Study approval

Blood samples were obtained from Gustave Roussy (Villejuif, France) and Saint Louis Hospital (Paris, France), with agreement from the Comité de Protection des Personnes Ile de France IV institutional review board (agreement from US Department of Health and Human Services: n°IRB 00003835-Protocol 2015/59-NICB) and Commission Nationale de l’Informatique et des Libertés (authorization #915663). Written informed consent forms were obtained in accordance with the Declaration of Helsinki.

Animal experiments were conducted in the Gustave Roussy animal facility and approved by the Gustave Roussy review board, authorization for the use of animals for scientific purposes protocol #2016–104 and project titled “Treatment and evolution of MPN.”

### Statistical analysis

In vivo data are presented as mean ± SEM and were analyzed with the Dunnett method as part of one-way ANOVA with multiple comparison tests (GraphPad Prism) or with the two-tailed Student’s *t* test. In vitro data were analyzed using the Student’s *t* test and two-way ANOVA for clonogenic assays. Error bars represent the mean ± SEM. In all figures, *P < 0.05, **P < 0.01, ***P < 0.001, and ****P < 0.0001.

### Online supplemental material

Fig. S1 compares the effects of IFN and ATO on cells expressing—or not expressing—JAK2^V617F^. Fig. S2 depicts the MPN mouse model used in Fig. 3. Fig. S3 compares senescence induction in control and JAK2^V617F^-expressing MPN cells.
